# Sex chromosome-to-autosome transposition events counter Y-chromosome gene loss in mammals

**DOI:** 10.1186/s13059-015-0667-4

**Published:** 2015-05-28

**Authors:** Jennifer F Hughes, Helen Skaletsky, Natalia Koutseva, Tatyana Pyntikova, David C Page

**Affiliations:** Whitehead Institute, Cambridge, MA 02142 USA; Howard Hughes Medical Institute, Whitehead Institute, Cambridge, MA 02142 USA; Department of Biology, Massachusetts Institute of Technology, Cambridge, MA 02142 USA

## Abstract

**Background:**

Although the mammalian X and Y chromosomes evolved from a single pair of autosomes, they are highly differentiated: the Y chromosome is dramatically smaller than the X and has lost most of its genes. The surviving genes are a specialized set with extraordinary evolutionary longevity. Most mammalian lineages have experienced delayed, or relatively recent, loss of at least one conserved Y-linked gene. An extreme example of this phenomenon is in the Japanese spiny rat, where the Y chromosome has disappeared altogether. In this species, many Y-linked genes were rescued by transposition to new genomic locations, but until our work presented here, this has been considered an isolated case.

**Results:**

We describe eight cases of genes that have relocated to autosomes in mammalian lineages where the corresponding Y-linked gene has been lost. These gene transpositions originated from either the X or Y chromosomes, and are observed in diverse mammalian lineages: occurring at least once in marsupials, apes, and cattle, and at least twice in rodents and marmoset. For two genes - *EIF1AX/Y* and *RPS4X/Y* - transposition to autosomes occurred independently in three distinct lineages.

**Conclusions:**

Rescue of Y-linked gene loss through transposition to autosomes has previously been reported for a single isolated rodent species. However, our findings indicate that this compensatory mechanism is widespread among mammalian species. Thus, Y-linked gene loss emerges as an additional driver of gene transposition from the sex chromosomes, a phenomenon thought to be driven primarily by meiotic sex chromosome inactivation.

**Electronic supplementary material:**

The online version of this article (doi:10.1186/s13059-015-0667-4) contains supplementary material, which is available to authorized users.

## Background

Although the mammalian X and Y chromosomes derive from the same autosomal ancestor, they are highly divergent in their present-day forms. The most pronounced contrast is in gene content: the Y chromosome has lost nearly all of the approximately 640 genes it once shared with the X chromosome [1]. A recent study comparing the ancestral regions of the Y chromosome across eight mammals (human, chimpanzee, rhesus macaque, marmoset, mouse, rat, cattle, and opossum) revealed that Y-chromosome loss was not a random process. Instead, the 36 genes that survived form a specialized set, functioning as gene regulators at multiple levels: chromatin modification, transcription, splicing, translation, and protein degradation [1]. Numerous lines of evidence indicate that these surviving genes, and their X-linked counterparts, are also more dosage sensitive than the remainder of the X chromosome’s ancestral genes, implying the influence of selective pressure to retain two copies of these genes in both sexes [1].

**Fig. 1:**
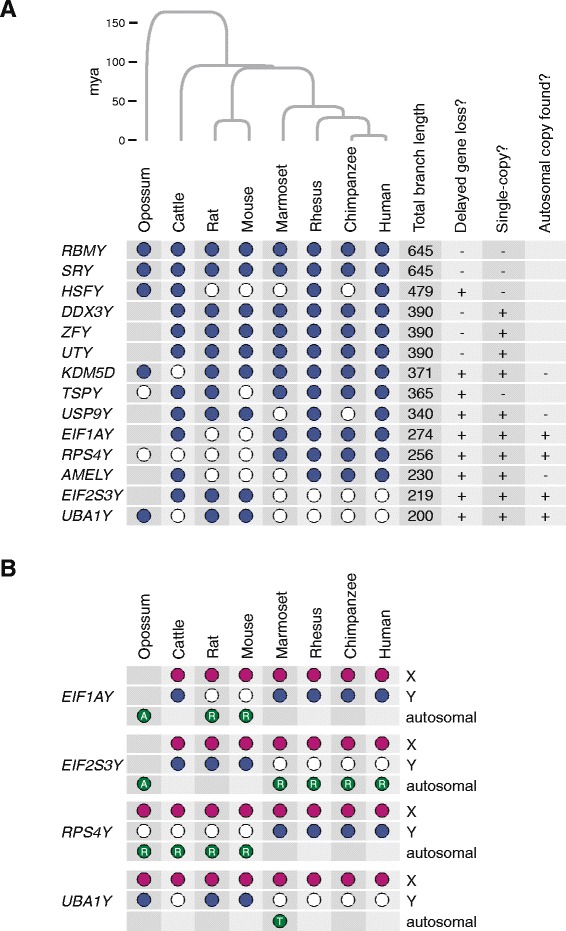
Distribution of long-lived Y-linked genes and their X-linked and autosomal homologs in eight mammals. **a** Species distribution and characteristics of the 14 longest-lived mammalian Y-linked genes. Phylogenetic tree representing evolutionary relationships among eight mammals is shown at top. Branch lengths are proportional to lineage divergence times; time scale in millions of years (mya) at left. The presence of a Y-linked gene in a given species is indicated by a blue circle; gene loss events are indicated by white circles. Blank squares in the opossum lineage represent seven genes that became Y-linked after the divergence of the eutherian and marsupial lineages. Genes are ranked according to total branch length, which is the sum of branch lengths for each species possessing an intact Y-linked homolog of that gene. The seven genes that were considered for further study met both of the criteria shown at the right side of the table: loss from the Y chromosome in one or more lineages and single-copy presence on the Y chromosome. Autosomal copies were found in one or more species for four of the seven genes. **b** Distribution of X-linked, Y-linked, and autosomal homologs of four genes in eight mammals. Filled circles indicate presence of gene in given species: X-linked (pink), Y-linked (blue), or autosomal (green). Autosomal copies are either ancestral (A), retrotransposed (R), or translocated (T), as indicated. White circles represent gene loss events

Most of the 36 ancestral genes endured on the Y chromosome for remarkably long time-spans and are shared across multiple mammalian lineages [1]. However, there are some glaring exceptions. Of the 14 most long-lived ancestral genes, nine genes were lost, relatively recently, in at least one mammalian lineage, and at least six of those genes were lost independently in multiple lineages over the course of mammalian evolution (Fig. 1a) [1]. Either these lost genes became expendable in certain lineages, which seems unlikely given their high degree of conservation, or gene loss was accompanied by a compensatory genetic mutation. One extreme example of a compensation mechanism that evolved to cope with Y-linked gene loss can be found in the Ryukyu spiny rat (*Tokudaia osimensis*). This species, which is indigenous to a single island in Japan, has no Y chromosome, but at least four ancestral Y chromosome genes (*EIF2S3Y*, *KDM5D*, *ZFY*, and *TSPY*) have been maintained in the genome because they were transposed to an autosome or to the X chromosome [2, 3].

Here we report that the rescue of Y-linked genes via transposition is widespread among mammals. We describe four genes - *EIF1A*, *EIF2S3*, *RPS4*, and *UBA1 -* whose lineage-specific Y-linked gene loss was accompanied by eight lineage-specific gene transposition events in multiple mammals (Fig. 1b). We present, on a case-by-case basis, the evolutionary history of the autosomal copies of the four sex-linked genes as well as evidence for their status as active genes.

## Results and discussion

We performed a systematic search of the genomes of the eight species included in the ancestral Y-chromosome comparison to identify autosomal homologs of long-lived genes that were recently lost from the Y chromosome in certain lineages [1]. We focused our analysis on seven genes that are single copy on the Y chromosome: *AMELY*, *EIF1AY*, *EIF2S3Y*, *KDM5D*, *RPS4Y*, *UBA1Y*, and *USP9Y* (Fig. 1a). We found lineage-specific transpositions countering Y loss for four of these genes: *EIF1AY*, *EIF2S3Y*, *RPS4Y*, and *UBA1Y* (Fig. 1b)*.* For the remaining three genes - *AMELY*, *KDM5D*, and *USP9Y* - no autosomal homologs were found in any species. Retrotransposition, which involves a processed mRNA intermediate, is a frequent occurrence in mammalian genomes and usually generates non-functional pseudogenes [4], so we used several criteria to evaluate the functionality of retrotransposed genes, or retrogenes. We only considered loci that met both of the following criteria: maintenance of an intact open reading frame (ORF) compared with its sex-linked counterpart (Additional file 1), and evidence for transcriptional activity using publically available RNA-seq datasets (Additional file 2). We dismissed retrogenes with very recent origins as judged by <2 % nucleotide divergence from their parental copy. These brand new retrogenes have not yet been subjected to culling by natural selection, so their functional relevance is dubious.

**Fig. 2:**
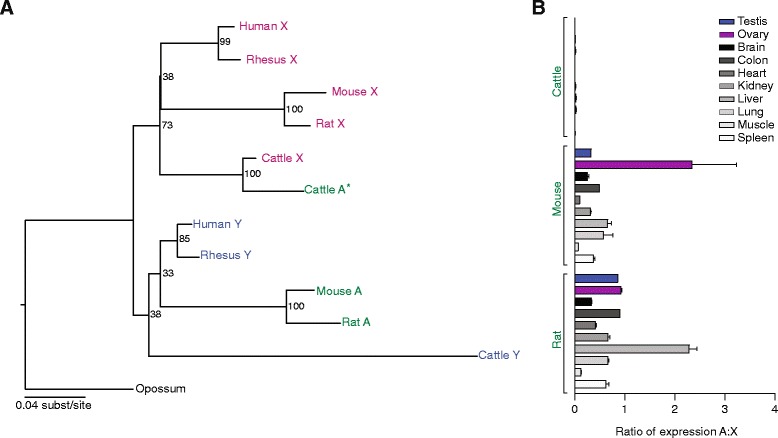
Evolution and expression patterns of *EIF1A* gene family members*.* **a** Maximum likelihood phylogenetic analyses of nucleotide coding sequences of X-linked (pink), Y-linked (blue), and autosomal (green) homologs. Asterisks indicate sequences with truncated ORFs. **b** RNA-seq analyses comparing expression levels of autosomal and X-linked homologs across various tissues. Means and standard errors are plotted

*EIF1A* (eukaryotic translation initiation factor 1) is located in the X/Y-added region, which is found in eutherian mammals but not marsupials [5]. *EIF1AY* is conserved among all major branches of the eutherian tree, with the exception of the rodent lineage (Fig. 1) [1, 6]. However, mouse and rat both contain an intact and actively transcribed autosomal *EIF1A* retrogene (Figs. 1b and 2b; Additional files 1, 2 and 3). Of the species retaining Y-linked copies of this gene, we found a retrotransposed copy in cattle, but it has a slightly truncated ORF and is transcribed at barely detectable levels compared with its X-linked homolog (Fig. 2b; Additional file 1). We therefore consider cattle autosomal *EIF1A* to be a pseudogene. Phylogenetic analysis clearly indicates that the rodent retrogene originated from the Y-linked copy of *EIF1A* (Fig. 2a)*.* This retrogene shows robust expression across multiple tissues, often matching and sometimes exceeding the expression of the X-linked copy (Fig. 2b). The mouse and rat retrogenes are located in syntenic genomic loci (Additional file 4), dating the origination of this retrogene to >25 million years ago (mya), and the dN/dS ratio of the mouse and rat retrogenes is 0.0010, indicating the action of strong purifying selection (Additional file 5). Taken together, the evidence strongly supports the conclusion that the *EIF1A* autosomal retrogene in mouse and rat serves as a substitute for *EIF1AY*.

**Fig. 3:**
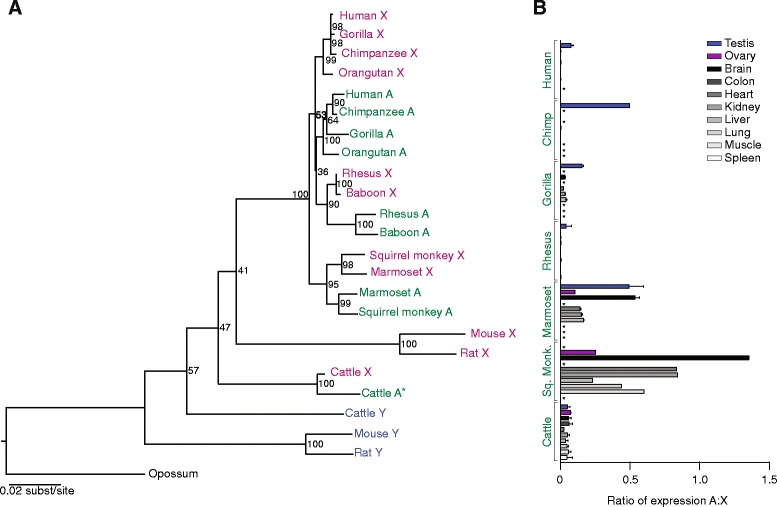
Evolution and expression patterns of *EIF2S3* gene family members*.* **a** Maximum likelihood phylogenetic analyses of nucleotide coding sequences of X-linked (pink), Y-linked (blue), and autosomal (green) homologs. Asterisks indicate sequences with truncated ORFs. **b** RNA-seq analyses comparing expression levels of autosomal and X-linked homologs across various tissues. Means and standard errors are plotted. Asterisks indicate no data for a given species/tissue

*EIF2S3* (eukaryotic translation initiation factor 2 subunit 3) is also located in the X/Y-added region and is conserved in all major branches of the eutherian tree except simian primates (Fig. 1) [1, 6, 7]. This absence is particularly curious given the finding that *Eif2s3y* plays a crucial role in mouse spermatogenesis [8]. A previous study reported an autosomal *EIF2S3* copy with testis-specific expression in human [7]. We found autosomal retrotranposed copies of *EIF2S3* in all primates examined, although in the Old World monkeys (OWMs: rhesus macaque and baboon) we could not confirm the integrity of the ORF because of gaps in the reference genome assemblies (Additional file 1). In contrast to *EIF1A* in rodents, phylogenetic analysis indicates that the *EIF2S3* retrogenes originated from the X-linked homolog (Fig. 3a). The *EIF2S3* retrogenes are found in different genomic locations in each of the three primate groups - in New World monkeys (NWMs: marmoset and squirrel monkey), in OWMs, and in apes (human, chimpanzee, gorilla, and orangutan) (Additional file 4), indicating that they arose independently at least three times during primate evolution, in agreement with phylogenetic analysis (Fig. 3a). Comparing the most divergent retrogene copies - human and squirrel monkey - the dN/dS ratio is 0.075, indicating strong purifying selection (Additional file 5). In our analysis, the retrogenes display very different expression patterns among the primate groups, with testis-specific or testis-predominant expression seen in apes and OWMs and expression across multiple tissues seen in NWMs (Fig. 3b; Additional files 2 and 3). This observation implies that, in apes and OWMs, the retrogene may have adopted a male-specific function. In NWMs, by contrast, the retrogene appears to have retained the broader function of the X-linked copy. Notably in cattle, where *EIF2S3Y* is retained, a relatively new retrotransposed copy of *EIF2S3* is found, but it has a truncated ORF, indicating pseudogenization (Fig. 3a; Additional file 1).

**Fig. 4:**
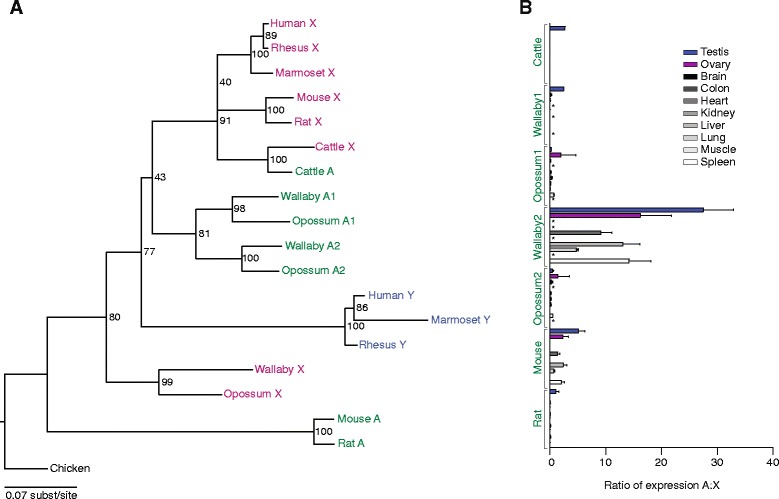
Evolution and expression patterns of *RPS4* gene family members. **a** Maximum likelihood phylogenetic analyses of nucleotide coding sequences of X-linked (pink), Y-linked (blue), and autosomal (green) homologs. **b** RNA-seq analyses comparing expression levels of autosomal and X-linked homologs across various tissues. Means and standard errors are plotted. Asterisks indicate no data for a given species/tissue

*RPS4* (ribosomal protein S4) is located in the evolutionarily conserved region of the sex chromosomes, so it was present on the Y chromosome of the eutherian-marsupial common ancestor. This ancient Y-linked gene is conspicuously absent in a number of mammals - mouse, rat, cattle, and opossum - representing three distinct lineages (Fig. 1) [1]. However, intact and active autosomal retrogenes are present in each of these species, and there are two *RPS4* retrogenes in opossum. Again, phylogenetic analysis indicates that this species distribution is the result of multiple, independent retrotransposition events, but it is unclear if the parental gene was the X or Y homolog in each case (Fig. 4a). The mouse and rat retrogenes are syntenic (Additional file 4), dating their origination at >25 mya. We also found syntenic copies of each of the two opossum retrogenes in wallaby (Additional file 4), which indicates that these loci are very ancient, originating >88 mya. Pairwise dN/dS calculations for mouse-rat, opossum1-wallaby1, opossum2-wallaby2, and opossum2-cattle are 0.0059, 0.0132, 0.0053, and 0.0180, respectively, providing strong evidence of purifying selection acting on the *RPS4* retrogenes in each lineage (Additional file 5). Surprisingly, *RPS4X* in wallaby contains an ORF-disrupting mutation, confirmed by RNA-seq analysis, so the two autosomal retrogenes are the only functioning *RPS4* copies in this species. While the marsupial retrogene copies have retained broad expression patterns, in rodents and cattle expression of the *RPS4* retrogene appears to be testis-specific or testis-predominant (Fig. 4b; Additional files 2 and 3). This implies the adoption of a new, male-specific function by the retrogene, which is reminiscent of the situation in primates where a duplicated, intron-containing Y-linked copy of *RPS4Y* displays testis-specific expression [9, 10]*. RPS4X* is yet another example of an X-linked ribosomal protein gene relocating to an autosome, a phenomenon that was previously reported in human for the X-linked genes *RPL10*, *RPL36A*, and *RPL39* [11].

**Fig. 5:**
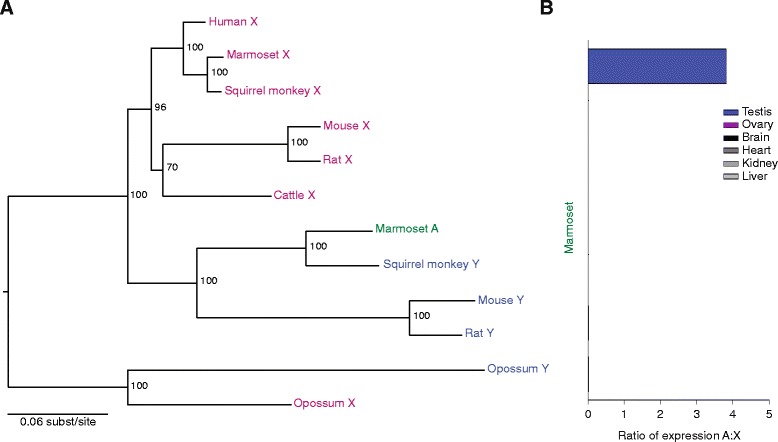
Evolution and expression patterns of *UBA1* gene family members. **a** Maximum likelihood phylogenetic analyses of nucleotide coding sequences of X-linked (pink), Y-linked (blue), and autosomal (green) homologs. **b** RNA-seq analyses comparing expression levels of autosomal and X-linked homologs across various tissues. Means and standard errors are plotted

The final example, *UBA1* (ubiquitin-like modifier activating enzyme 1), was present on the Y chromosome of the eutherian-marsupial ancestor, like *RPS4*, and it is missing or inactivated in a number of species - notably cattle and most primates (Fig. 1) [1, 12, 13]. We found an autosomal copy of *UBA1* in only one species - marmoset. This autosomal copy arose via genomic transposition, not retrotransposition, from the Y chromosome. The transposed region is roughly 68 kb in size, and the autosomal *UBA1* gene contains introns and is flanked by Y-chromosome-derived sequence. Interestingly, another NWM - the squirrel monkey - has retained *UBA1* on the Y chromosome [13], and this is closely related to the autosomal copy in marmoset (Fig. 5a; dN/dS = 0.3381; Additional file 5). Because their Y chromosomes are not completely sequenced, we experimentally confirmed the autosomal and Y-chromosomal locations of these genes in marmoset and squirrel monkey, respectively (Additional file 6). Expression of the marmoset autosomal *UBA1* is strictly testis-specific among the tissues examined (Fig. 5b; Additional files 2 and 3), just as the Y-linked gene in mouse, rat, and opossum appears to be testis-specific (Additional files 2 and 3). The male-specific function of this gene thus appears to be uniquely essential in NWMs among the primates.

We analyzed expression data for a total of 16 transposed genes across 11 species (Figs. 2b, 3b, 4b and 5b). Nine of the 16 transposed genes exhibit broad expression patterns (evidence of transcription in multiple tissues) similar to their X-linked counterparts (Figs. 2b, 3b, 4b and 5b). In these nine cases, selection to preserve a lower bound of gene dosage in a broad range of tissues may have been the driving force behind this phenomenon. We note that the expression levels of the broadly expressed transposed genes relative to their X-linked counterparts varies widely between tissues and between species. We infer that selective pressures to adjust gene expression levels may operate differently among tissues and species. To assess the trend of broad expression of sex-chromosome-to-autosome transposed genes on a global level, we evaluated the expression patterns of all human retrogenes originating from the X chromosome (25 total) using publically available RNA-seq data, as well as the expression patterns of their X-linked progenitors. In 13 cases, the breadth of expression [1] of the autosomal retrogene was equivalent to or greater than the breadth of expression of the corresponding X-linked gene in both sexes (Additional file 7).

By contrast, 7 of the 16 transposed genes reported here are expressed predominantly if not exclusively in the testis among the tissues examined (Figs. 2b, 3b, 4b and 5b). Most of these testis-specific transposed genes - *EIF2S3* in human, chimpanzee, and rhesus, and *RPS4* in cattle, mouse, and rat - have become functionally specialized, as their X- and Y-linked counterparts are broadly expressed in all other species. By contrast, the testis-specific transposed copy of *UBA1* in marmoset has apparently retained its ancestral function, as its Y-linked counterpart is also testis-specific in the species in which it is found. One additional case of a testis-specific gene that relocated from the Y chromosome to an autosome has been reported: *HSFY*, which is found in eutherians and marsupials and is multi-copy in human, rhesus macaque, and cattle, is autosomal in rodents, where it retains a testis-specific expression pattern [14, 15]. Taken all together, these findings indicate that, upon Y-linked gene loss, sex-chromosome-derived retrogenes may be maintained on the autosomes either to preserve a lower bound of gene dosage or to preserve male-specific function.

## Conclusions

**Fig. 6:**
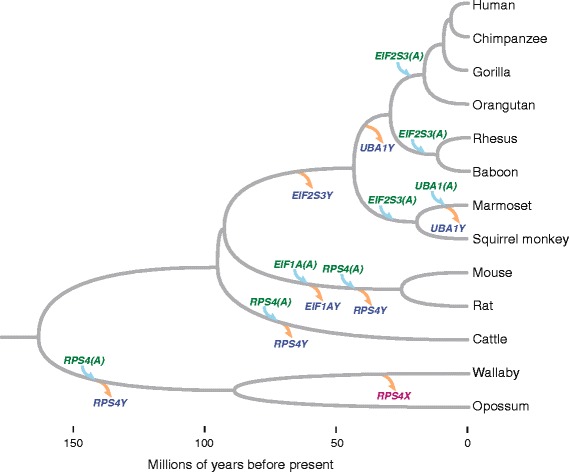
Examples of gene transposition to autosomes compensating for delayed, lineage-specific loss of homologous Y-chromosome genes: a phylogenetic perspective. Phylogenetic tree representing evolutionary relationships among mammalian species analyzed in the current study. Branch lengths are proportional to lineage divergence times; time scale in millions of years is shown at bottom. Gene loss and addition events are placed on particular branches of the tree according to species distribution patterns; precise timing of events is not known. Gene loss events (Y-linked gene loss or X-linked gene loss) are indicated by orange arrows; gene addition events (autosomal retrogene formation or gene transposition) are indicated by blue arrows. Gene names are color-coded to indicate chromosomal location: Y-linked (blue), autosomal (green), and X-linked (pink)

Until now, the rescue of delayed Y-linked gene loss via gene transposition was thought to be a peculiarity found in an isolated rodent species, but the new data presented here indicate that this compensatory mechanism is widespread among mammals. We found eight cases of sex chromosome-to-autosome gene transposition events that counter delayed, lineage-specific loss of the corresponding Y-linked genes (Fig. 6). These events occurred in every major branch of the mammalian tree examined, and, for two genes (*RPS4X/Y* and *EIF2S3X/Y*), independent retrotransposition events occurred at least three times. Furthermore, there is strong evidence that the transposed autosomal genes deriving from the sex chromosomes have been effectively preserved by natural selection over long evolutionary time spans, indicating that these new genes most likely have important biological functions. In some cases, the new gene copies have broad expression patterns, like their sex-linked counterparts, and may function to preserve a lower bound of gene dosage that is essential for fitness. This situation is relevant when the X- and Y-linked copies of a gene encode proteins that have redundant functions. Relocation to an autosome may or may not result in an initial, possibly deleterious, increase in gene dosage, depending on the promoter and enhancer elements present in the new genomic location. In other cases, the transposed genes appear to exhibit testis-specific expression patterns, suggesting that the autosomal copies have diverged in function from their sex-linked counterparts and have acquired a male-specific function, perhaps in spermatogenesis.

Previous studies of genome-wide retrogene distribution in human have found a bias towards origination from the X chromosome. The presumptive rationale for this elevated 'out-of-X' pattern is the drive to escape meiotic sex chromosome inactivation (MSCI), which transcriptionally silences sex-linked genes during male meiosis [16–18]. MSCI is thoroughly studied only in mouse [19], but is thought to be a process common to all eutherians [16]. It is speculated that essential spermatogenesis genes were forced to relocate to an autosome upon the advent of MSCI in mammals, and the enrichment of testis-biased expression patterns among retrogenes lends support to this hypothesis [20, 21]. Our re-analysis of expression patterns of human X-to-autosome retrogenes indicates that, in roughly half of the cases, the retrogene displays a broad expression pattern, not the testis-specific expression pattern predicted by the MSCI hypothesis (Additional file 7). Therefore, an alternative explanation for this phenomenon is required. Here we have identified a novel selective force that contributes to transposition from the X (and Y) chromosome - rescue of Y-linked gene loss. It is also possible that the two scenarios are not mutually exclusive: MSCI may have selected for a sex-chromosome-to-autosome retrogene, and once the transposed gene is established, selection to maintain the Y-linked copy is relaxed. Reference-grade X- and Y-chromosome sequences from additional mammalian species will enable a more complete understanding of the impact of sex-chromosome-to-autosome transposition events on genome evolution as well as the selective pressures driving this phenomenon.

## Materials and methods

### Phylogenetic analyses

Nucleotide sequence alignments were generated using MUSCLE [22], and phylogenetic trees, with 100 bootstrap replicates, were generated using PhyML [23]. GenBank accession numbers for gene sequences are provided in Additional file 1. dN/dS ratios were calculated using PAL2NAL [24]. Species divergence dates are from the TimeTree database [25].

### RNA-seq analyses

RNA-seq datasets were downloaded from GenBank; accession numbers are provided in Additional file 2. For each species, reads were mapped (using Bowtie [26]) to autosomal and X-linked homologs of a given gene. Ratios of numbers of reads mapping to autosomal versus X-linked homologs, normalized by length, were calculated.

### Fluorescence *in situ* hybridization

Fluorescence *in situ* hybridization (FISH) was performed as previously described [27] on male marmoset fibroblasts obtained from the Sam and Ann Barshop Institute for Longevity and Aging Studies (animal ID 113/17043).

### PCR

Male and female squirrel monkey genomic DNA samples were obtained from Coriell Institute (ID PR00474: *Saimiri boliviensis* and NG05311: *Saimiri sciureus*, respectively). Two sets of *UBA1Y*-specific primers were used: 5′-AATCCTTGCTTGCCTCACTG-3′ + 5′-GGTGGCCTGCTATGTTGACT-3′; 5′-AGGATGCAACAGAGGTAGTGA-3′ + 5′-CCAGGCCTCCAATGAAAGC-3′.

## Additional files

Additional file 1: Table S1. Chromosomal locations, open reading frame positions, and GenBank accession numbers for *EIF1A*, *EIF2S3*, *RPS4*, and *UBA1* gene family members. Additional file 2: Table S2.
**(A)** List of RNA-seq datasets used in gene expression analyses with GenBank accession numbers and sexes of tissues indicated. **(B)** Tabular results of RNAseq analyses for all species, tissues, and X, Y, and autosomal gene family members. Additional file 3: Figure S1. Scatter plots of RNAseq analyses (from **Table S2B**) for all species, tissues, and X, Y, and autosomal gene family members. Female (F) and male (M) data are shown separately. Testes shown in blue, ovary shown in magenta, and all other somatic tissues shown in gray (see **Table S2B** for complete list). FPKM = fragments mapping to gene per kilobase gene length per millions of fragments in given dataset. Python script used to generate plots - betavioplot.py - is available at [28]. Additional file 4: Figure S2. Genomic locations of transposed autosomal genes. Screen shots from USCS Genome Browser [29] showing genomic locations of retrogenes and neighboring genes in various species to demonstrate synteny of retrogenes. **(A)** Mouse and rat *Eif1a* retrogene. **(B)** Human and orangutan *EIF2S3* retrogene. **(C)** Rhesus and baboon *EIF2S3* retrogene. **(D)** Marmoset and squirrel monkey *EIF2S3* retrogene. **(E)** Mouse and rat *Rps4* retrogene. **(F)** Opossum and wallaby *RPS4* retrogene (only one of the two *RPS4* retrogenes is shown because the second wallaby retrogene is located in a short sequence contig that contains no neighboring genes). Additional file 5: Table S3. Synonymous and non-synonymous substitution rate (dS and dN) calculations. Additional file 6: Figure S3. Confirmation of *UBA1* localization in New World monkeys. **(A)** FISH analysis on male marmoset (*Callitrix jacchus*) cells. Green signal corresponds to *UBA1*; red signal is specific to Y chromosome. **(B)** PCR analysis on male (M) and female (F) squirrel monkey (*Saimiri sciureus*) genomic DNA using two sets of *UBA1Y*-specific primers. Additional file 7: Table S4.
**(A)** Expression breadth of all human X-to-autosome retrogenes and their X-linked progenitors. Female and male data are shown separately. **(B)** FPKM values used to calculate expression breadth.

## Abbreviations

FISH: fluorescence *in situ* hybridization
MSCI: meiotic sex chromosome inactivation
mya: million years ago
NWM: New World monkey
ORF: open reading frame
OWM: Old World monkey
PCR: polymerase chain reaction
