# Supplementary figures and images for: Sex chromosome-to-autosome transposition events counter Y-chromosome gene loss in mammals
Source: Genome Biol. 2015 May 28;16(1):104. doi: 10.1186/s13059-015-0667-4 (PMC4446799; doi:10.1186/s13059-015-0667-4)

Figure S1

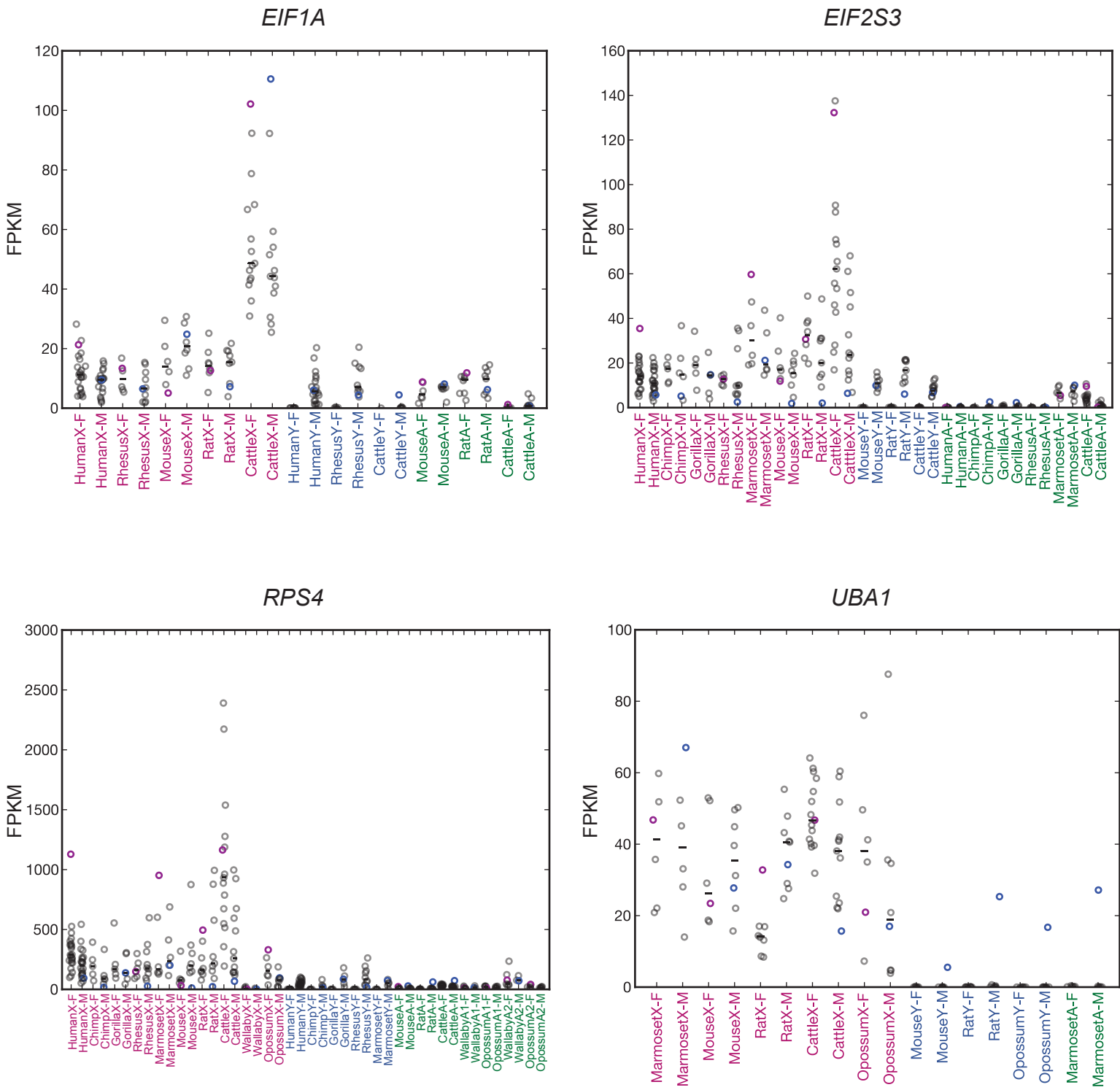

Supplement: Additional file 3: Figure S1. — Scatter plots of RNAseq analyses (from Table S2B) for all species, tissues, and X, Y, and autosomal gene family members. Female (F) and male (M) data are shown separately. Testes shown in blue, ovary shown in magenta, and all other somatic tissues shown in gray (see Table S2B for complete list). FPKM = fragments mapping to gene per kilobase gene length per millions of fragments in given dataset. Python script used to generate plots - betavioplot.py - is available at [28]. [file 13059_2015_667_MOESM3_ESM.pdf]

Figure S3

**A** Marmoset

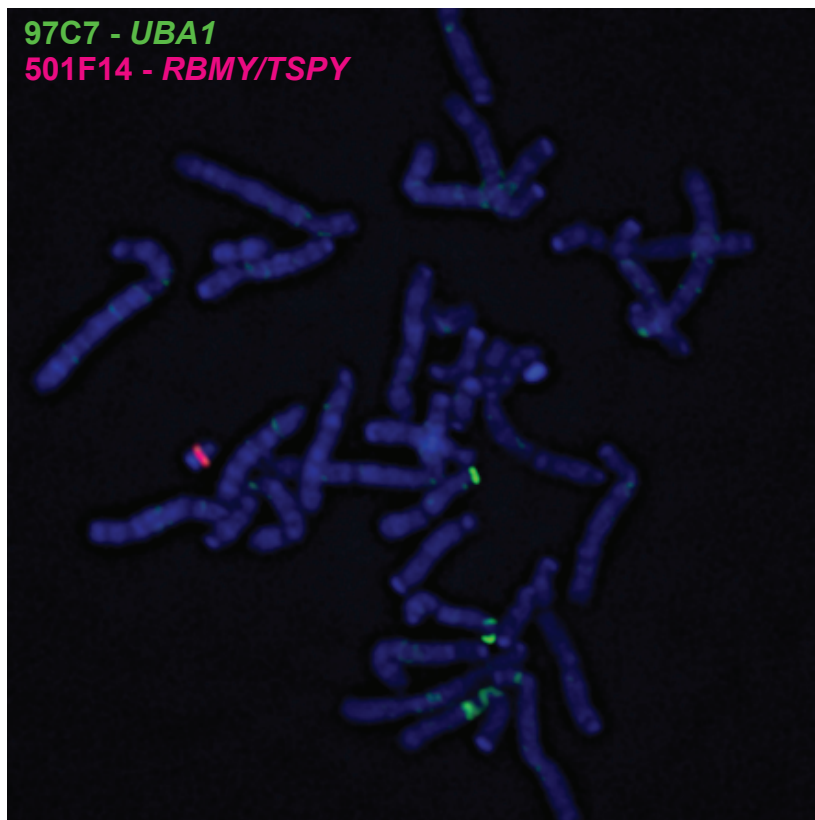

**B** Squirrel monkey

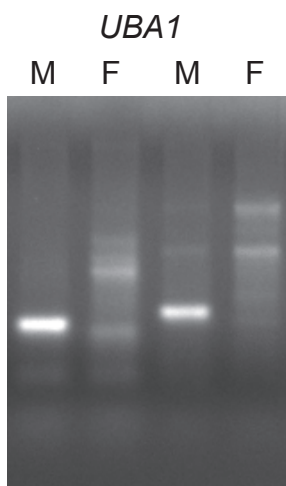

Supplement: Additional file 6: Figure S3. — Confirmation of UBA1 localization in New World monkeys. (A) FISH analysis on male marmoset (Callitrix jacchus) cells. Green signal corresponds to UBA1; red signal is specific to Y chromosome. (B) PCR analysis on male (M) and female (F) squirrel monkey (Saimiri sciureus) genomic DNA using two sets of UBA1Y-specific primers. [file 13059_2015_667_MOESM6_ESM.pdf]
