# Supplementary material for: Sex chromosome-to-autosome transposition events counter Y-chromosome gene loss in mammals
Source: Genome Biol. 2015 May 28;16(1):104. doi: 10.1186/s13059-015-0667-4 (PMC4446799; doi:10.1186/s13059-015-0667-4)
Supplement: Additional file 4: Figure S2. — Genomic locations of transposed autosomal genes. Screen shots from USCS Genome Browser [29] showing genomic locations of retrogenes and neighboring genes in various species to demonstrate synteny of retrogenes. (A) Mouse and rat Eif1a retrogene. (B) Human and orangutan EIF2S3 retrogene. (C) Rhesus and baboon EIF2S3 retrogene. (D) Marmoset and squirrel monkey EIF2S3 retrogene. (E) Mouse and rat Rps4 retrogene. (F) Opossum and wallaby RPS4 retrogene (only one of the two RPS4 retrogenes is shown because the second wallaby retrogene is located in a short sequence contig that contains no neighboring genes). [file 13059_2015_667_MOESM4_ESM.pdf]

Figure S2

A

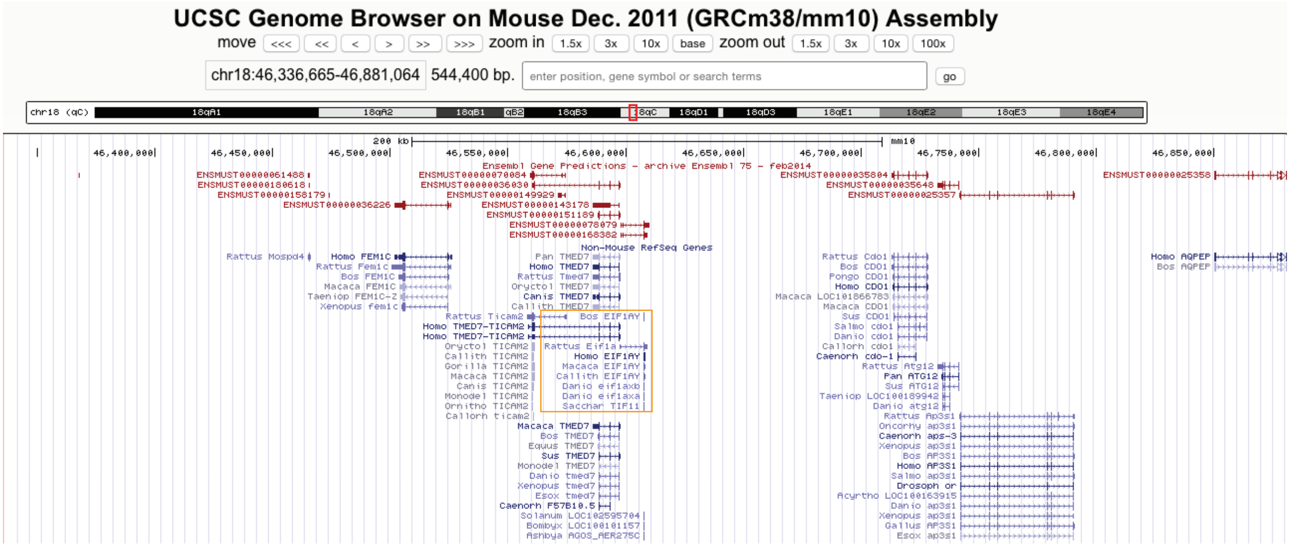

UCSC Genome Browser on Mouse Dec. 2011 (GRCm38/mm10) Assembly
move <<< << < > >> >>> zoom in 1.5x 3x 10x base zoom out 1.5x 3x 10x 100x
chr18:46,336,665-46,881,064 544,400 bp.
enter position, gene symbol or search terms
go
chr18 (qC)
18qA1 18qA2 18qB1 qB2 18qB3 18qC 18qD1 18qD3 18qE1 18qE2 18qE3 18qE4
200 kb
mm10
46,400,000 46,450,000 46,500,000 46,550,000 46,600,000 46,650,000 46,700,000 46,750,000 46,800,000 46,850,000
Ensembl Gene Predictions - archive Ensembl 75 - feb2014
Non-Mouse RefSeq Genes

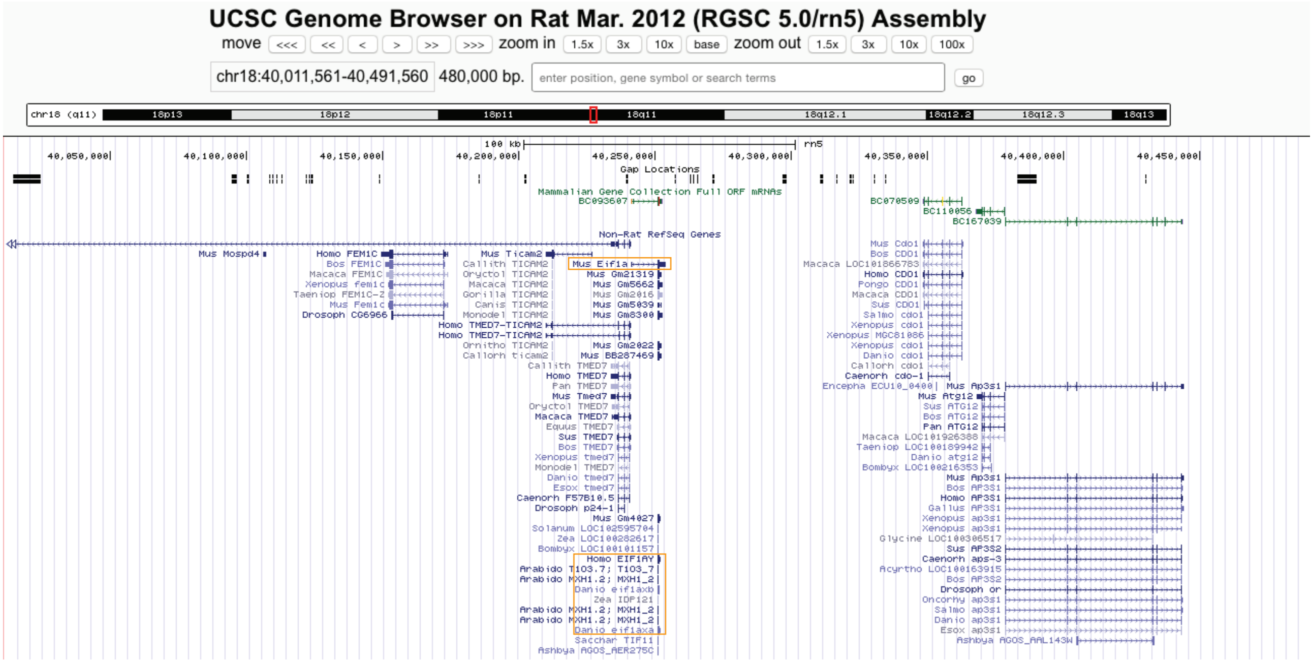

UCSC Genome Browser on Rat Mar. 2012 (RGSC 5.0/rn5) Assembly
move <<< << < > >> >>> zoom in 1.5x 3x 10x base zoom out 1.5x 3x 10x 100x
chr18:40,011,561-40,491,560 480,000 bp.
enter position, gene symbol or search terms
go
chr18 (q11)
18p13 18p12 18p11 18q11 18q12.1 18q12.2 18q12.3 18q13
100 kb
rn5
40,050,000 40,100,000 40,150,000 40,200,000 40,250,000 40,300,000 40,350,000 40,400,000 40,450,000
Gap Locations
Mammalian Gene Collection Full ORF mRNAs
Non-Rat RefSeq Genes

Figure S2
B

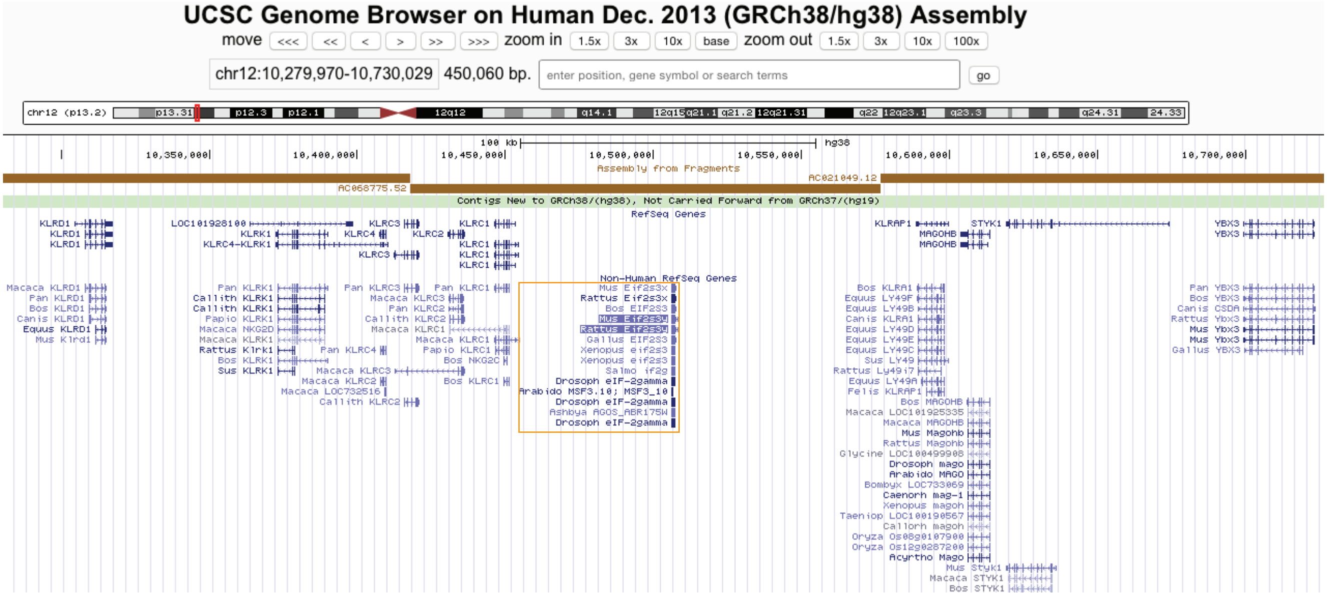
UCSC Genome Browser on Human Dec. 2013 (GRCh38/hg38) Assembly
move <<< << < > >> >>> zoom in 1.5x 3x 10x base zoom out 1.5x 3x 10x 100x
chr12:10,279,970-10,730,029 450,060 bp.
enter position, gene symbol or search terms
go
chr12 (p13.2)
100 kb hg38
10,350,000 10,400,000 10,450,000 10,500,000 10,550,000 10,600,000 10,650,000 10,700,000
Assembly from Fragments
AC068775.52
AC021049.12
Contigs New to GRCh38/(hg38), Not Carried Forward from GRCh37/(hg19)
RefSeq Genes
Non-Human RefSeq Genes

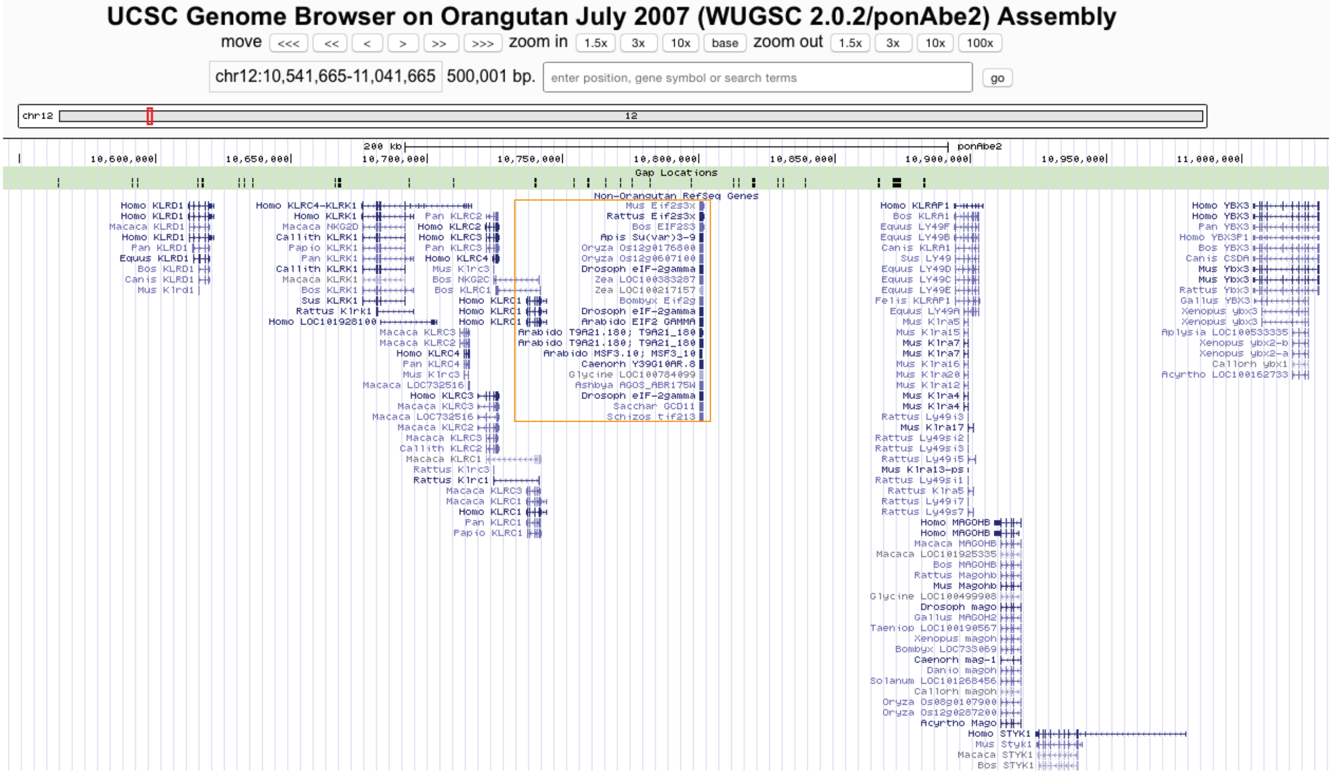
UCSC Genome Browser on Orangutan July 2007 (WUGSC 2.0.2/ponAbe2) Assembly
move <<< << < > >> >>> zoom in 1.5x 3x 10x base zoom out 1.5x 3x 10x 100x
chr12:10,541,665-11,041,665 500,001 bp.
enter position, gene symbol or search terms
go
chr12
200 kb ponAbe2
10,600,000 10,650,000 10,700,000 10,750,000 10,800,000 10,850,000 10,900,000 10,950,000 11,000,000
Gap Locations
Non-Orangutan RefSeq Genes

Figure S2
C

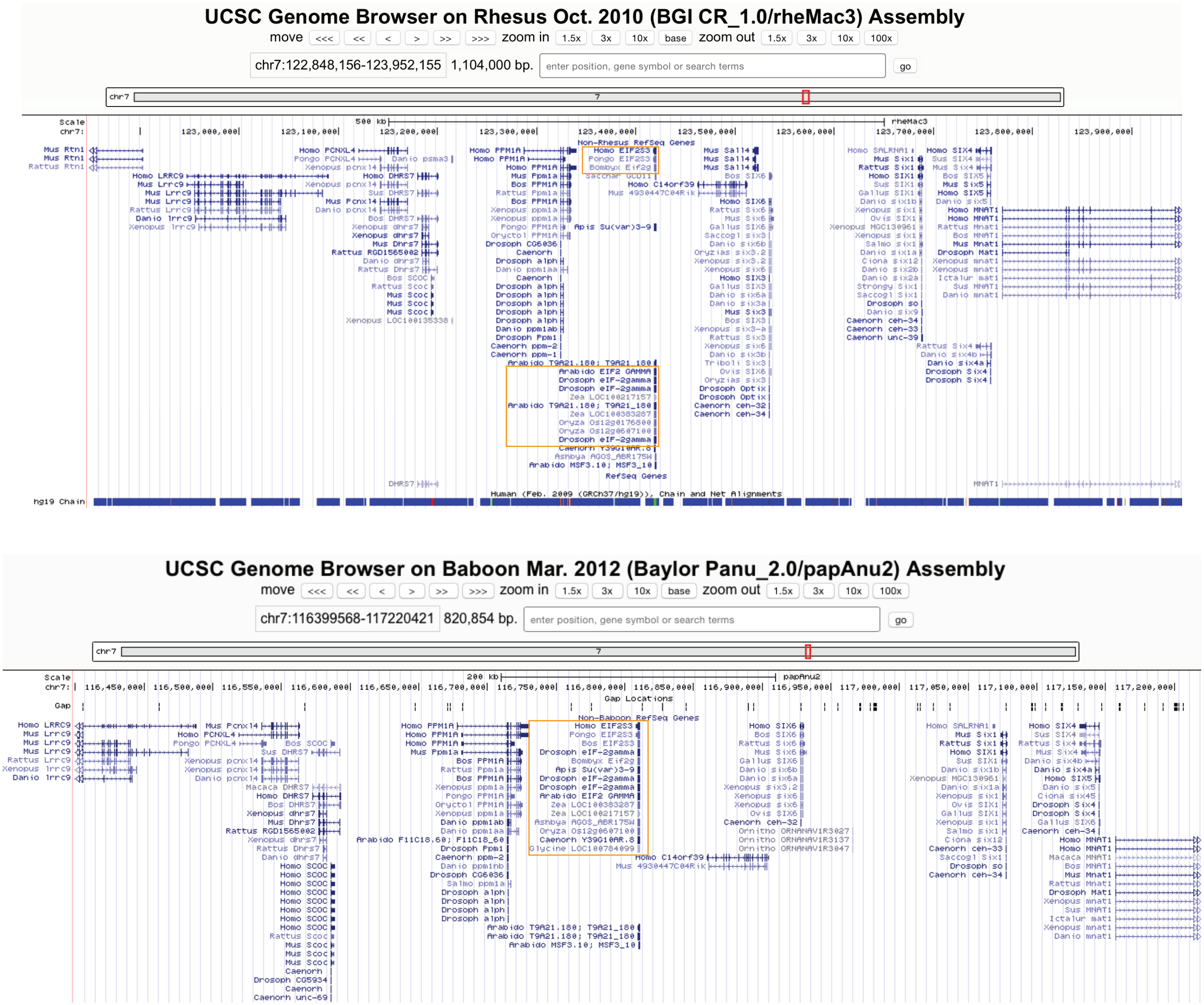
UCSC Genome Browser on Rhesus Oct. 2010 (BGI CR_1.0/rheMac3) Assembly
move <<< << < > >> >>> zoom in 1.5x 3x 10x base zoom out 1.5x 3x 10x 100x
chr7:122,848,156-123,952,155 1,104,000 bp.
enter position, gene symbol or search terms
go
Non-Rhesus RefSeq Genes
RefSeq Genes
Human (Feb. 2009 (GRCh37/hg19)), Chain and Net Alignments
hg19 Chain
UCSC Genome Browser on Baboon Mar. 2012 (Baylor Panu_2.0/papAnu2) Assembly
move <<< << < > >> >>> zoom in 1.5x 3x 10x base zoom out 1.5x 3x 10x 100x
chr7:116399568-117220421 820,854 bp.
enter position, gene symbol or search terms
go
Gap Locations
Non-Baboon RefSeq Genes

Figure S2

D

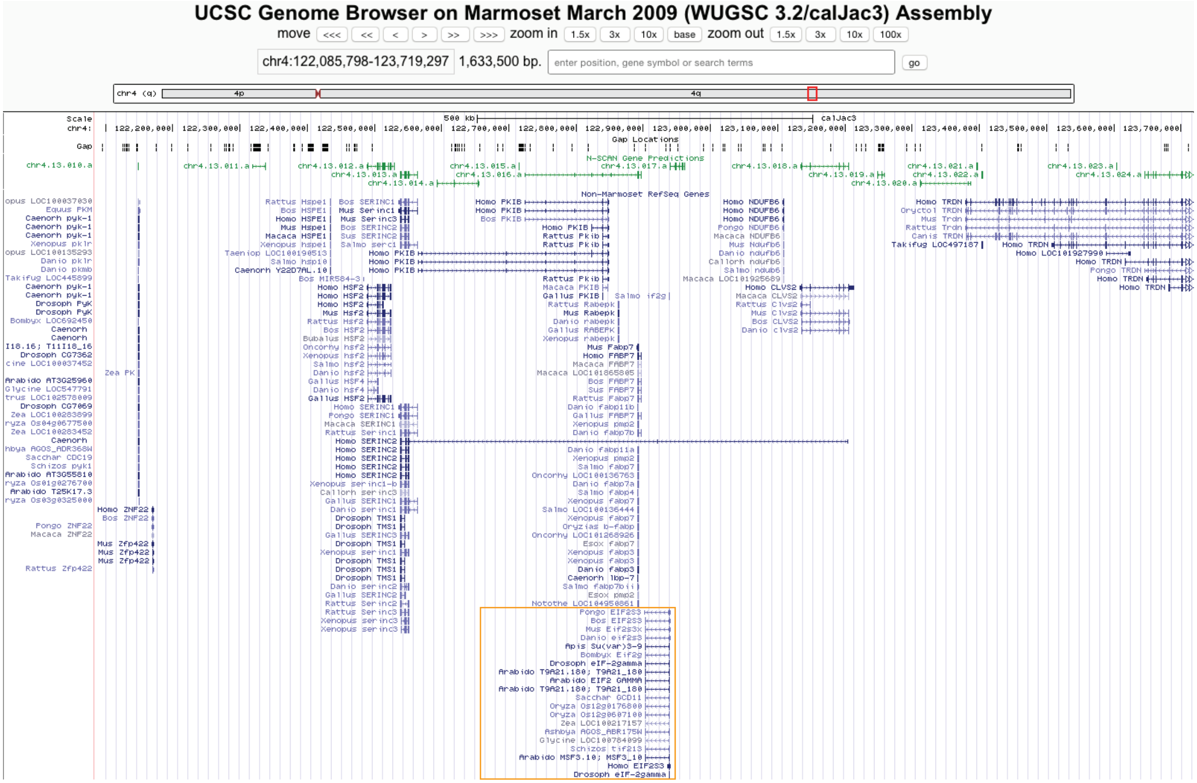
UCSC Genome Browser on Marmoset March 2009 (WUGSC 3.2/calJac3) Assembly
move <<< << < > >> >>> zoom in 1.5x 3x 10x base zoom out 1.5x 3x 10x 100x
chr4:122,085,798-123,719,297 1,633,500 bp.
enter position, gene symbol or search terms
go
chr4 (q)
4p
4q
Gap Locations
N-SCAN Gene Predictions
Non-Marmoset RefSeq Genes

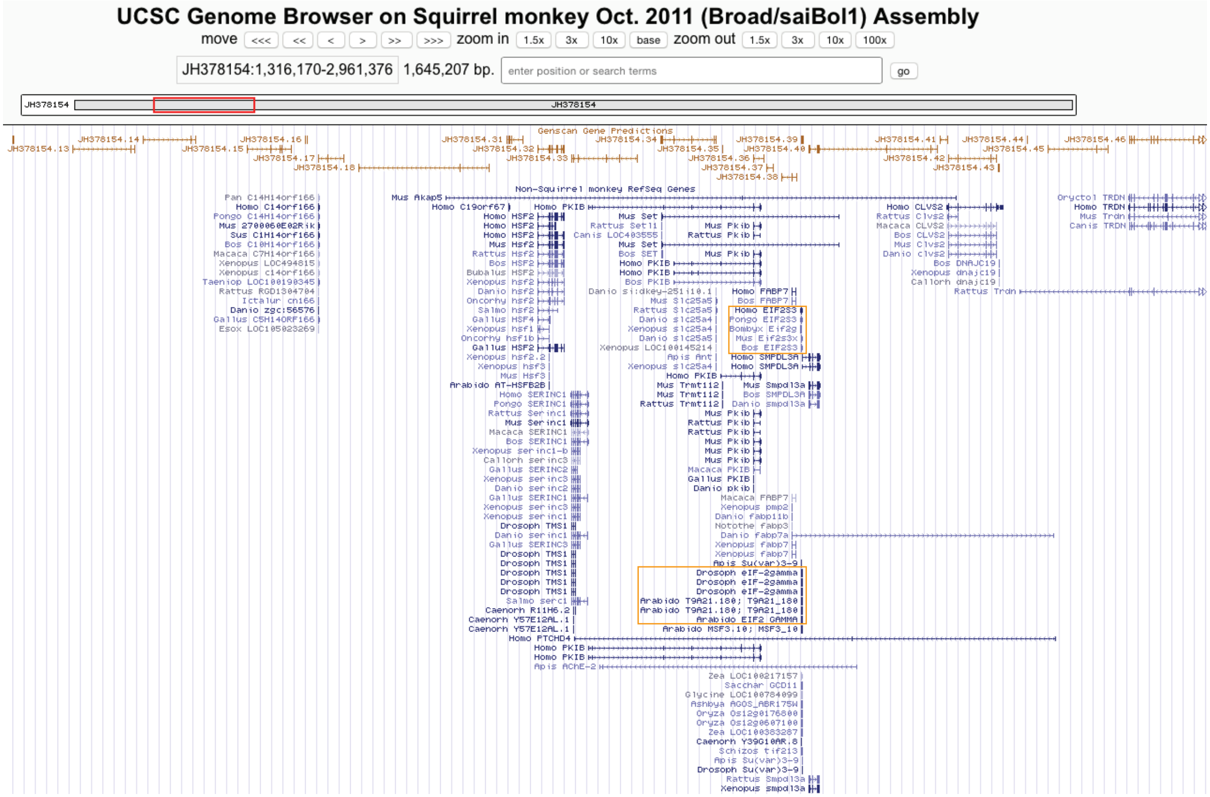
UCSC Genome Browser on Squirrel monkey Oct. 2011 (Broad/saiBol1) Assembly
move <<< << < > >> >>> zoom in 1.5x 3x 10x base zoom out 1.5x 3x 10x 100x
JH378154:1,316,170-2,961,376 1,645,207 bp.
enter position or search terms
go
JH378154
Genscan Gene Predictions
Non-Squirrel monkey RefSeq Genes

Figure S2

E

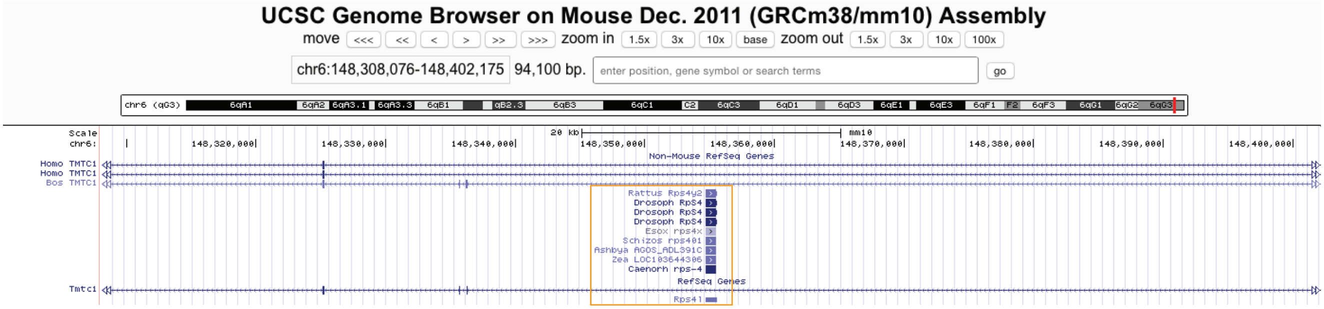
UCSC Genome Browser on Mouse Dec. 2011 (GRCm38/mm10) Assembly
move
zoom in
zoom out
chr6:148,308,076-148,402,175 94,100 bp.
enter position, gene symbol or search terms
go
Non-Mouse RefSeq Genes
RefSeq Genes

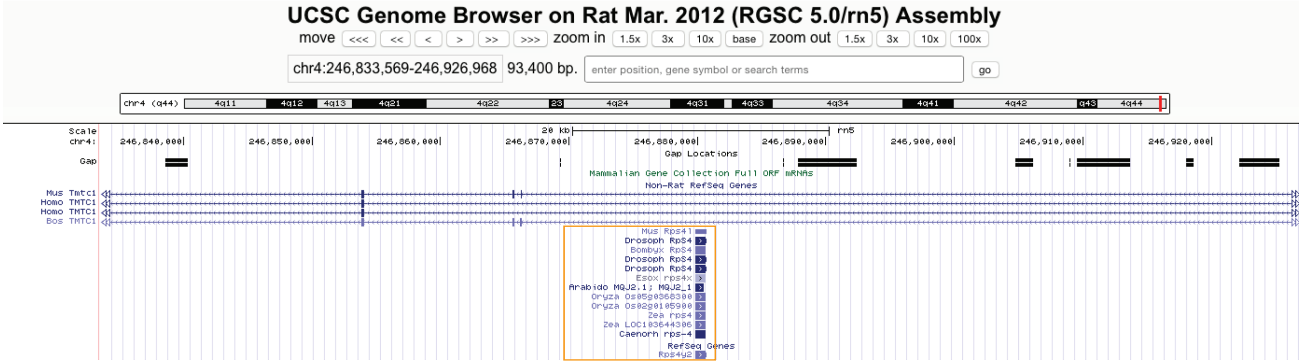
UCSC Genome Browser on Rat Mar. 2012 (RGSC 5.0/rn5) Assembly
move
zoom in
zoom out
chr4:246,833,569-246,926,968 93,400 bp.
enter position, gene symbol or search terms
go
Gap Locations
Mammalian Gene Collection Full ORF mRNAs
Non-Rat RefSeq Genes
RefSeq Genes

Figure S2

F

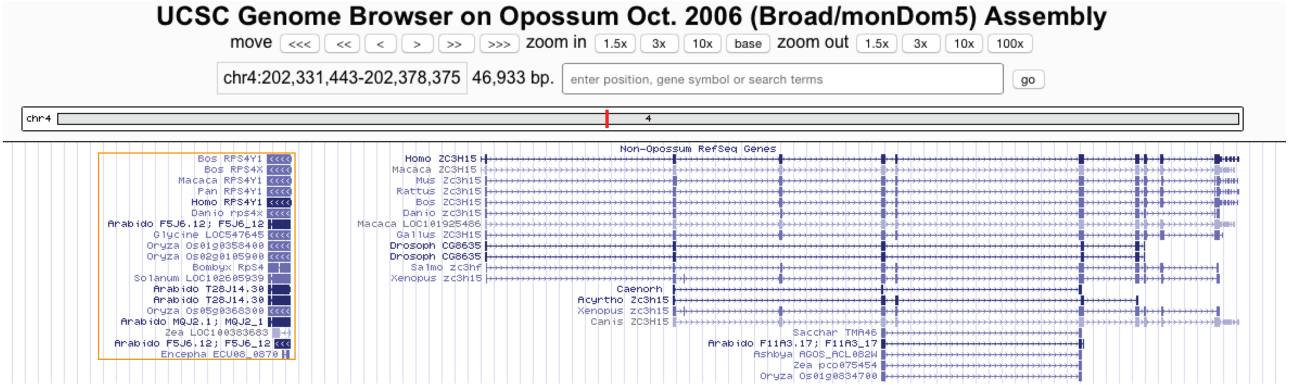

UCSC Genome Browser on Opossum Oct. 2006 (Broad/monDom5) Assembly
move <<< << < > >> >>> zoom in 1.5x 3x 10x base zoom out 1.5x 3x 10x 100x
chr4:202,331,443-202,378,375 46,933 bp.
enter position, gene symbol or search terms
go
chr4
4
Non-Opossum RefSeq Genes
Bos RPS4Y1
Bos RPS4X
Macaca RPS4Y1
Pan RPS4Y1
Homo RPS4Y1
Danio rps4x
Arabido F5J6.12; F5J6_12
Glycine LOC547645
Oryza Os01g0358400
Oryza Os02g0105900
Bombyx RpS4
Solanum LOC102605939
Arabido T28J14.30
Arabido T28J14.30
Oryza Os05g0368300
Arabido MQJ2.1; MQJ2_1
Zea LOC100383683
Arabido F5J6.12; F5J6_12
Encepha ECU08_0870
Homo ZC3H15
Macaca ZC3H15
Mus Zc3h15
Rattus Zc3h15
Bos ZC3H15
Danio zc3h15
Macaca LOC101925486
Gallus ZC3H15
Drosoph CG8635
Drosoph CG8635
Salmo zc3hf
Xenopus zc3h15
Caenorh
Acyrtho Zc3h15
Xenopus zc3h15
Canis ZC3H15
Sacchar TMA46
Arabido F11A3.17; F11A3_17
Ashbya AGOS_ACL082W
Zea pco075454
Oryza Os01g0834700

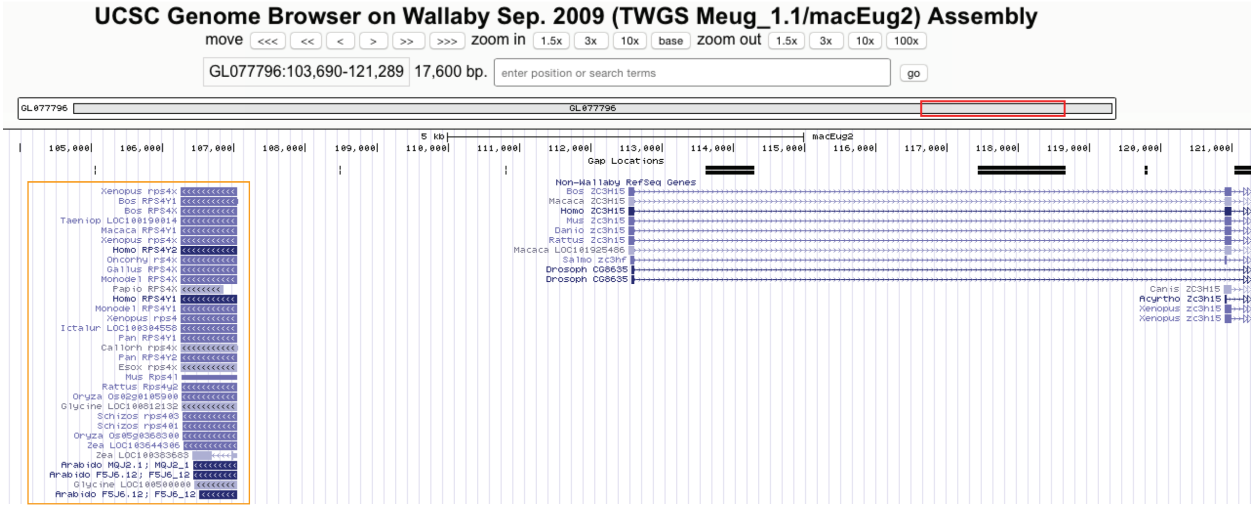

UCSC Genome Browser on Wallaby Sep. 2009 (TWGS Meug_1.1/macEug2) Assembly
move <<< << < > >> >>> zoom in 1.5x 3x 10x base zoom out 1.5x 3x 10x 100x
GL077796:103,690-121,289 17,600 bp.
enter position or search terms
go
GL077796
GL077796
5 kb
macEug2
105,000
106,000
107,000
108,000
109,000
110,000
111,000
112,000
113,000
114,000
115,000
116,000
117,000
118,000
119,000
120,000
121,000
Gap Locations
Non-Wallaby RefSeq Genes
Xenopus rps4x
Bos RPS4Y1
Bos RPS4X
Taeniop LOC100190014
Macaca RPS4Y1
Xenopus rps4x
Homo RPS4Y2
Oncorhy rs4x
Gallus RPS4X
Monodel RPS4X
Papio RPS4X
Homo RPS4Y1
Monodel RPS4Y1
Xenopus rps4
Ictalur LOC100304558
Pan RPS4Y1
Callorh rps4x
Pan RPS4Y2
Esox rps4x
Mus Rps4l
Rattus Rps4y2
Oryza Os02g0105900
Glycine LOC100812132
Schizos rps403
Schizos rps401
Oryza Os05g0368300
Zea LOC103644306
Zea LOC100383683
Arabido MQJ2.1; MQJ2_1
Arabido F5J6.12; F5J6_12
Glycine LOC100500000
Arabido F5J6.12; F5J6_12
Bos ZC3H15
Macaca ZC3H15
Homo ZC3H15
Mus Zc3h15
Danio zc3h15
Rattus Zc3h15
Macaca LOC101925486
Salmo zc3hf
Drosoph CG8635
Drosoph CG8635
Canis ZC3H15
Acyrtho Zc3h15
Xenopus zc3h15
Xenopus zc3h15
